# How do patients with primary hypertension assess different endpoints of their treatment? a survey using analytic hierarchy process

**DOI:** 10.1038/s41371-026-01135-8

**Published:** 2026-03-23

**Authors:** Charalabos-Markos Dintsios, Nadezda Chernyak

**Affiliations:** Medical Faculty, Institute for Health Services Research and Health Economics, Centre for Health and Society, Heinrich-Heine-University Düsseldorf, Düsseldorf, Germany

**Keywords:** Health care, Renovascular hypertension

## Abstract

Effects of antihypertensive therapy are estimated in clinical trials. There is a need to prioritize the endpoints according to patients’ preferences. 26 patients from two regions of Germany rated in 2019 their preferences regarding the importance of various endpoints of hypertension treatment (Mortality, Myocardial infarction, Stroke, Heart failure, and subdivided Adverse events) by a pairwise comparison of individual endpoints. Analytic Hierarchy Process (AHP), a multi-criteria decision analysis method was used to generate relative weights for each endpoint. The robustness of the results was defined by means of consistency. The elicitation yielded the following aggregated group weights: Stroke 0.320, Mortality 0.297, Myocardial infarction 0.202, Heart failure 0.119, and Adverse events 0.062, subdivided in Dyspnea, Pain, Edema, and Cough. The overall consistency reached for efficacy endpoints a consistency ratio below 0.1 (safety endpoints = 0.04) without exceeding established limits. In all sensitivity analyses but one, no rank reversal was observed, and Stroke was rated highest. Individual weights varied extensively. Some participants weighted Mortality (0.021–0.686) higher than Stroke (0.078–0.615) and Heart failure (0,021–0,469) higher than Myocardial infarction (0,047–0.431). Individual inconsistency exceeded the limits in almost half of the cases, with gender, therapy duration, and therapeutic scheme being explaining variables for inconsistency within binary logistic regression models. AHP can be used to obtain preferences of patients with primary hypertension for effectiveness and safety endpoints. Preference elicitation could provide important information for drug assessment (group weights) and shared decision-making (individual weights) following the concept of patient-centeredness at system and patient level.

## Introduction

*Hypertension* is the most significant controllable risk factor for cardiovascular diseases, accounting for an estimated 57% of all cardiovascular-related deaths [1, 2]. Moreover, *hypertension* is strongly associated with an increased risk of *Stroke*, *Myocardial infarction*, and all-cause *Mortality*. The guidelines of the European Society of Cardiology (ESC) recommend different drug classes with evidence on clinical outcomes in the target population (ACE inhibitors, ARBs, dihydropyridine CCBs, diuretics, and beta-blockers) to achieve a target of blood pressure-lowering treatment of 120 – 129/70 – 79 mmHg [3].

*Patient-centeredness* in health policy is increasingly recognized as a major value policy makers wish to adhere to. It requires a solid evidence-base for patient-related issues in healthcare and patient-oriented assessment of health interventions. *Patient preference* studies may contribute to that evidence base.

Among several methods that have been used to elicit *patient preferences* concerning *endpoints*, Analytic Hierarchy Process (AHP), a technique for multi-criteria decision analysis (MCDA) [4], fulfills the respective required features. It has been shown that the AHP works well to support a variety of health care decisions [5, 6]. The AHP assists patients and health care professionals in grasping complex decisions by guiding them to prioritize and has been applied to facilitate a wide variety of health care decisions with multiple, even competing, outcomes [7].

Unlike available patient preference studies targeting clinical endpoints for chronic conditions such as rheumatoid arthritis [8], chronic hepatitis C [9] and diabetes mellitus [10], there are no studies eliciting endpoint preferences in patients with primary hypertension. The primary aim of the study was to investigate how patients suffering from high blood pressure prioritize *efficacy* and *safety endpoints* for the assessment of pharmaceutical interventions in *primary hypertension* treatment.

## Methods

### Trial design

In this cross-sectional study, patients suffering from high blood pressure completed a self-reported AHP survey on *efficacy* and *safety endpoints* of pharmaceutical antihypertensive treatment. (Sub-)Criteria of the survey were gathered by systematic literature search and validated within a focus group. The survey was pre-tested by the same focus group.

### Participants and recruitment

The inclusion criteria for participation were defined as i) a diagnosis of *primary hypertension* and ii) current use of medication for its treatment – regardless of the number of medications taken daily – as well as the presence of iii) sufficient language skills and iv) cognitive abilities. Furthermore, patients had to be v) of adult age. Exclusion criteria were defined as i) other forms of *hypertension* and ii) further (multi-)morbidity in the cardiovascular and cerebrovascular area, to obtain statements on patients’ endpoint weighting triggered solely by *primary hypertension*. The patients should iii) not have suffered a cardio- or cerebrovascular event, i.e. not be treated with antihypertensives for secondary prevention, to avoid biased endpoint weightings based on their own experience of the event. This did not apply to *Adverse events*, which are medication-related and thus within the experience of any patient receiving anti-hypertensive medication.

The survey setting for the application of the AHP questionnaire as part of the structured interview consisted of three pharmacies (one in North-Rhine-Westfalia and two in Bavaria) to guarantee regional representativeness. Face-to-face interviews were conducted in a separate customer area, the existence of which is common in German pharmacies. A convenience sampling was followed from March to June 2019.

### Sample size

There is no established minimum sample size for AHP in the literature [11, 12]. A review of different AHP survey applications in healthcare depicted a range of one to 1300 participants [7]. We referred to a sample size in AHP surveys with healthcare relevance [13–18]. A target sample size of at least 25 participants was set to ensure a robust dataset for the AHP.

### Analytical hierarchy process

The Analytic Hierarchy Process (AHP) is a structured decision-making method designed to analyze complex problems by systematically incorporating subjective *preferences* into the decision-making process [7, 19]. AHP is classified as a compositional method within Multi-Criteria Decision Analysis (MCDA).

To implement AHP, an overarching goal is defined first, followed by the hierarchical structuring of decision criteria. Participants perform pairwise comparisons to evaluate the relative importance of criteria, using a standardized numerical rating scale (ESM Fig. 1). These comparisons provide the basis for calculating numerical weights for each criterion. Finally, the weighted scores are aggregated by maximum eigenvalue of the matrix of pairwise comparisons to determine the rankings of the criteria. For more details on the matrix algebra implemented in AHP see Dolan et al. 1989 [20] and Supplementary Material (ESM Text 1).

**Fig. 1:**
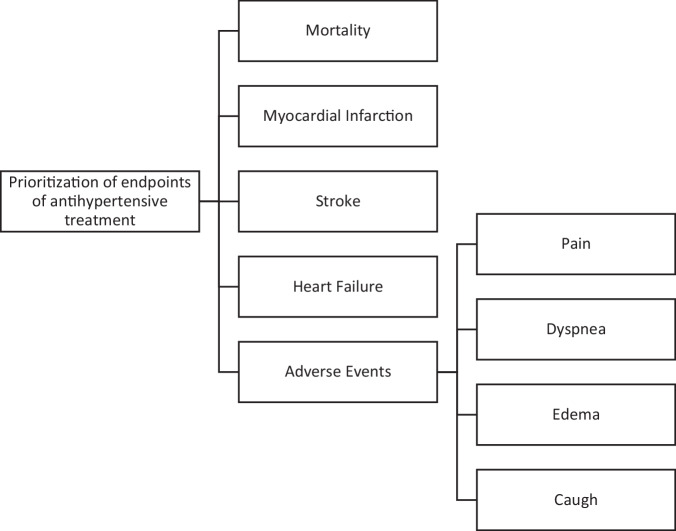
AHP structure. The figure shows the AHP decision structure with two levels: the 1st level includes the efficacy and aggregated safety endpoints, whereas the 2nd level refers to the individual safety endpoints.

The main criteria are categories of outcomes that are conceptually related (Fig. 1). Since only outcomes were prioritized by patients and *hypertension* treatments were not compared, the hierarchical levels only include in this case the objectives and (sub-)criteria without treatment alternatives. *Endpoints* were identified from a systematic literature review about different antihypertensive drugs as first line therapy in patients with *primary hypertension* [21] and from the interviews with patients when testing the feasibility and use of the developed specific AHP questionnaire in a respective focus group. The *endpoints* were selected according to the following rationale in respect to the methodological constraints: the *endpoints* needed to be mutually exclusive, clear, comprehensive, and to be of importance within the same order of magnitude. The literature review led to the selection of the following *endpoints*: *Mortality, Myocardial infarction, Heart failure, Stroke* and *Adverse events*, consistent with established clinical *endpoints* in *hypertension* research [3].

### Statistical analysis

The analysis comprised four phases. In the first phase the values of the weighted *efficacy endpoints* (first hierarchy level) and *safety endpoints* (second hierarchy level) were derived by the principal right eigenvector approach for each participating patient after completion of the AHP survey instrument. Thereafter, the consistency ratios were calculated to check for potential inconsistencies due to violation of one or more of the quality criteria for AHP, i.e., reflexivity, reciprocity, and transitivity on an individual level. In the next phase the aggregated weights were calculated again with the right eigenvector approach by averaging the individual results through geometric means and consistency was checked on an aggregated group level. In the third phase based on different stratification criteria (consistency and health related quality of life) subgroup analyses were implemented to check for robustness of the aggregated weights (extent and hierarchical order) and potential rank reversals. In the final (fourth) phase, the explorative binary logistic regression model was calculated to gain more insights on potential factors impacting the consistency of the *preference* weights. Statistical significance of the model and its coefficients as well as explainable variance were determined. The binary regression model was chosen, since the dependent variable is per definition dichotomized based on a consistency threshold. Multicollinearity was tested by means of variance inflation factor (VIF). The principal right eigenvector was calculated using matrix multiplication including respective iterations and the generation of random matrices to derive the random index (R.I.) according to Dolan et al. [20] with MS excel [22]. The different regressions were conducted using the regression module of SPSS (version 16).

### Elicitation instrument

**Fig. 2:**
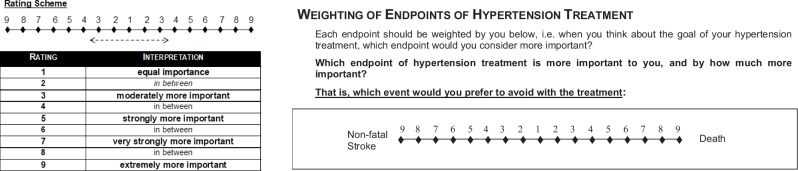
Excerpt from the AHP survey on endpoints. The excerpt includes exemplary one question asked to the participants regarding their pair-wise importance ratings when comparing respective endpoints.

The applied instrument consisted of a questionnaire developed specifically for the survey, which was administered to the participants using a structured interview. The structured interview primarily served to ensure compliance with the inclusion and exclusion criteria and to allow questions to the interviewer in case of (cognitive) overload of the participants. Postal or digital transmission of the questionnaire integrated into the structured interview was omitted to avoid missing data and the completion by third parties (family members or life partners). The *endpoints* were described in layman’s language. Various public patient information websites served as sources, primarily including the German Institute of Quality and Efficiency in Health Care (IQWiG) website https://www.informedhealth.org/. The description of the efficacy and safety endpoints is given in Supplementary Material (ESM Text 2). The interviewer was trained to convey consistent content in relation to the relevant descriptions. First, before using the survey instrument, it was tested in a focus group session with five affected patients regarding (i) required language skills, (ii) cognitive effort, (iii) provided background information, and (iv) the participants’ potential willingness to weigh up options. Second, to ensure a common understanding of the pairwise comparisons, participants were asked to repeat the introductory example and clarifying explanation were offered in case of misconceptions. Excerpts of the questionnaire used in the AHP survey, and the AHP rating scheme are presented in Fig. 2.

## Results

### Demographics

Of the 28 patients who gave their consent, only the data of 26 patients could be analyzed, as one patient failed for cognitive reasons despite the interviewer’s accompanying survey and another patient refused to participate in the corresponding endpoint assessments. 87 patients contacted refused to give their consent due to time constraints. The sociodemographic characteristics of the anonymized analyzed participants, as well as information on drug therapy and its duration, can be found in Table 1. The average age is 61 ± 12 years (50% male) with a treatment duration of 9.8 ± 6.9 years (65% of those received combination therapy).

**Table 1:** Baseline characteristics.

Nr.	Age (years)	Gender	Treatment duration (years)	Treatment scheme	VAS Utility (%)
1	72	m	20	mono	60
2	72	m	8	combi	60
3	69	m	18	combi	50
4	64	f	6	combi	75
5	86	f	21	combi	40
6	54	m	7	combi	80
7	67	f	20	combi	80
8	55	f	3	mono	95
9	45	m	1	mono	75
10	67	m	16	combi	70
11	58	m	20	mono	70
12	69	f	15	combi	50
13	53	f	10	mono	80
14	37	f	4	mono	70
15	77	m	22	combi	50
16	46	m	1	combi	65
17	66	m	7	combi	90
18	61	f	5	combi	90
19	71	m	11	combi	50
20	40	m	1	mono	80
21	43	f	2	mono	90
22	52	f	5	combi	80
23	63	f	10	mono	90
24	71	m	9	combi	90
25	69	f	10	combi	70
26	65	f	4	combi	80
Σ	61 ± 12	50%m	9.8 ± 6.9	65% combination	72.3 ± 15.3

Health-related quality of life (HRQoL) was used as a discriminatory criterion. The visual analogue scale (VAS) from the validated and standardized generic index instrument EQ-5D-3Lwas used for this purpose. The average utility values (in %) of the analyzed survey population derived from the VAS was 72.31 ± 15.31 (Table 1). 23% (6/26) of the participants stated a VAS utility greater than or equal to 90, which is considered to be a very high utility (almost ceiling effect). On the other hand, 27% (7/26) stated a rather low VAS utility of less than or equal to 60. The patient characteristics indicate that participants were suffering from *primary hypertension* of varying severity, but all of them were event-naive regarding cerebrovascular and cardiovascular events.

### AHP survey

The AHP procedure yielded the following aggregated group results for *preference-based* weighting of the *endpoints* surveyed (global weight) in descending order (Table 2): 1. *Stroke* (0.320). 2. *Mortality* (0.297). 3. *Myocardial infarction* (0.202). 4. *Heart failure* (0.119). 5. *Adverse events* of the therapies (0.062), subdivided again (global weight, local weight) in 5a. *Pain* (0.020, 0.316), 5b. *Cough* (0.008, 0.122), 5c. *Dyspnea* (0.024, 0.397), and 5 d. *Edema* (0.010, 0.165).

**Table 2:** Individual and group weights and consistency.

**Nr**.	**CR Endpoints**	**CR Adverse Events**	**Mortality**	**Myocardial Infarction**	**Stroke**	**Heart Failure**	**Adverse events**	**Pain**	**Dyspnea**	**Cough**	**Edema**	**Individual-Wi**
1	1.49*	0.16	0.164	0.187	0.200	0.284	0.165	0.659	0.076	0.171	0.094	
2	0.13	0.15	0.510	0.146	0.266	0.036	0.042	0.394	0.408	0.061	0.137	
3	0.13	0.23	0.290	0.131	0.507	0.036	0.037	0.610	0.260	0.039	0.090	
4	0.14	0.08	0.417	0.125	0.395	0.031	0.031	0.698	0.182	0.060	0.060	
5	0.12	0.11	0.046	0.166	0.615	0.085	0.088	0.673	0.152	0.076	0.099	
6	0.78*	0.19	0.087	0.134	0.295	0.469	0.015	0.178	0.514	0.041	0.267	
7	0.12	0.23	0.207	0.095	0.501	0.149	0.049	0.188	0.605	0.091	0.116	
8	0.19	0.13	0.534	0.121	0.121	0.202	0.022	0.626	0.266	0.054	0.054	
9	0.01	1.11*	0.212	0.240	0.240	0.282	0.027	0.366	0.283	0.327	0.024	
10	0.00	0.00	0.686	0.078	0.078	0.080	0.078	0.250	0.250	0.250	0.250	
11	0.00	0.22	0.667	0.083	0.083	0.083	0.083	0.427	0.218	0.137	0.218	
12	0.28*	0.00	0.596	0.088	0.203	0.036	0.077	0.444	0.444	0.560	0.560	
13	0.48*	0.50*	0.325	0.121	0.448	0.089	0.018	0.225	0.675	0.075	0.025	
14	0.00	1.81*	0.243	0.243	0.243	0.243	0.027	0.355	0.190	0.166	0.289	
15	0.19	0.19	0.087	0.158	0.432	0.272	0.051	0.294	0.173	0.071	0.462	
16	0.20	0.12	0.574	0.121	0.203	0.075	0.027	0.150	0.509	0.070	0.271	
17	0.19	0.44*	0.130	0.259	0.428	0.146	0.037	0.033	0.665	0.216	0.086	
18	0.40*	1.23*	0.609	0.092	0.236	0.039	0.024	0.129	0.573	0.140	0.158	
19	0.20	0.38*	0.067	0.381	0.397	0.035	0.120	0.223	0.656	0.085	0.036	
20	0.33*	1.91*	0.622	0.088	0.229	0.021	0.040	0.285	0.337	0.018	0.359	
21	1.21*	0.50*	0.296	0.322	0.347	0.023	0.012	0.075	0.225	0.025	0.675	
22	0.72*	1.46*	0.021	0.431	0.213	0.152	0.183	0.299	0.398	0.056	0.246	
23	0.37*	0.94*	0.262	0.143	0.282	0.105	0.208	0.146	0.354	0.212	0.288	
24	0.57*	1.82*	0.041	0.240	0.231	0.212	0.275	0.173	0.314	0.210	0.303	
25	0.50*	0.88*	0.592	0.246	0.102	0.042	0.018	0.109	0.208	0.628	0.054	
26	0.41*	3.33*	0.608	0.047	0.222	0.104	0.019	0.252	0.324	0.249	0.175	
	**Endpoints**		**Mortality**	**Myocardial Infarction**	**Stroke**	**Heart Failure**	**Adverse Events**	**Pain**	**Dyspnea**	**Cough**	**Edema**	
	**Group-W**_**i**_	^**W**^**all**	0.297	0.202	0.320	0.119	0.062	0.316	0.397	0.122	0.165	
		^**W**^**cons**	0.303	0.182	0.351	0.104	0.060	0.359	0.373	0.121	0.147	

*) Consistency ratio > 0.2

*CR* consistency ratiom *W*_*i*_ weight i, *W*_*all*_ weight derived from all participants, *W*_*cons*_ weight derived only from consistent participants.

**Fig. 3:**
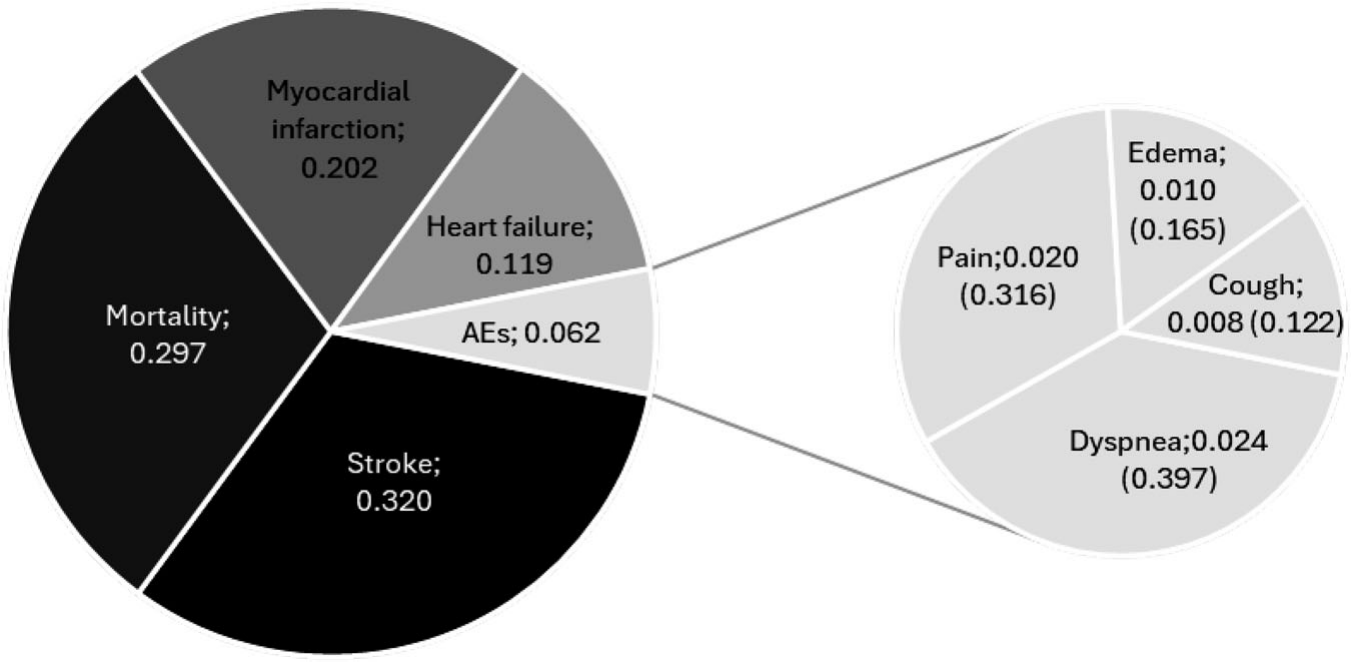
AHP results endpoint weights. The hierarchy tree contains the global weights for efficacy and aggregated safety endpoints at the 1st hierarchy level and the global (overarching criteria) and local weights (sub-criteria) for the individual safety endpoints at the 2nd hierarchy level.

The observed group inconsistency remained below the limit of 0.1 at both hierarchical levels. *Stroke* emerged as the most important endpoint, followed by *Mortality*. *Myocardial infarction* and the endpoint *Heart failure* followed. *Adverse events*, including severe ones such as *Dyspnea*, played a less important role from an aggregated group perspective. At the second hierarchy level, where *Adverse events* were differentiated and compared individually, *Dyspnea* predominated, followed by *Pain*. *Edema* and *Cough* played a less important role among respondents (Fig. 3).

On the other hand, individual weights varied extensively (Table 2). Some participants weighted Mortality (0.021–0.686) higher than Stroke (0.078–0.615) and Heart failure (0,021–0,469) higher than Myocardial infarction (0,047–0.431) deviating more or less from the aggregated group weighting order. Furthermore, a few participants weighted *Adverse events* (0.015 – 0.275) very highly, even exceeding the individual weights for *Mortality* or *Stroke*. At the second hierarchical level of the different Adverse events *Pain* (0.033 – 0.698), *Cough* (0.018 – 0.628), *Dyspnea* (0.076 – 0.675), and *Edema* (0.024 – 0.675) the results were heterogeneous with broad and overlapping ranges. As the participants were under antihypertensive treatment (mono or combination therapies), this mainly reflects their experience regarding *Adverse events* according to their feedback and the feedback of the focus group during the pre-test of the survey.

According to Saaty [11], an evaluation matrix is sufficiently consistent if the CR ≤ 0.1. Other authors, however, suggest for complex hierarchies and those that are not amenable to immediate modification during the evaluation process, a limit of 0.2 [23–25]. This was also the situation in the present analysis, as the CR could not be determined in parallel during the interview, which would have disrupted the interview process and jeopardized the survey in the chosen form.

### Subgroup analysis

When considering individually consistent weightings (CR ≤ 0.2), the following endpoint order resulted: *Stroke* 0.351, *Mortality* 0.303, *Myocardial infarction* 0.182, *Heart failure* 0.104, and *Adverse events* 0.060. The information on the respective individual consistency ratios and the comparison of the weights between the entire survey population and the consistent participants can be found in Table 2. Overall, 61% (16/26) of participants showed inconsistencies that exceeded the threshold of a CR ≤ 0.2 at least at one or both hierarchy levels. 11% (3/26) showed inconsistencies only at the first hierarchy level, 15% (4/26) only at the second level, and 34% (9/26) at both levels. Assuming that the first hierarchy level is more important for the research question than the second (weighting of the *Adverse events* relative to each other), 46% (12/26) were inconsistent at least at this level. The order of weighting did not change in the results obtained, but *Stroke* proved to be even more important for patients with consistent ratings.

In addition, subgroup analysis based on HRQoL was performed. Stratification regarding disease severity using HRQoL was undertaken based on the utility values from the VAS. The value of 75 on the VAS, which represents the median of the respondents’ results, was selected as the threshold for dichotomization into severe and less severe disease states. The analysis was carried out across all patients and subsequently exclusively across consistent patients (CR ≤ 0.2) at the first hierarchy level. Of the 26 patients included, 12 patients reported a VAS utility score of less than 75 (40–70). The remaining 14 patients showed a range of 75 to 95. The respective weights of the patients stratified according to the VAS utility scores are shown in Table 3.

**Table 3:** Preference weights stratified according to VAS utilities.

		Mortality	Myocardial Infarction	Stroke	Heart Failure	Adverse Events	C.R.	N
**VAS-Utility**	**VAS-U**_**<75**_ **All**	0.334	0.192	0.301	0.106	0.067	0.01	12
	**VAS-U**_**<75**_ **Consistent**	0.300	0.192	0.335	0.104	0.069	0.02	9
	**VAS-U**_**>75**_ **All**	0.286	0.193	0.335	0.132	0.055	0.03	14
	**VAS-U**_**>75**_ **Consistent**	0.300	0.178	0.338	0.149	0.035	0.07	5

*C.R*. consistency ratio.

In 3 of the 4 constellations (VAS-U < 75 consistent, VAS-U > 75 all and VAS-U > 75 consistent) *Stroke* receives a higher weight than *Mortality*. Only when considering all patients with a VAS utility value of less than 75 *Mortality* (0.334) received a higher weight than *Stroke* (0.301), which in turn cannot be maintained when only consistent weightings of the patients are included. The reason for this is the three times higher rating of *Mortality* compared to *Stroke* in two of the three inconsistent participants with a VAS utility of less than 75, who, as outliers, distort the overall result in this direction. The order of the other *endpoints* does not change depending on HRQoL compared to the total sample. It can therefore be concluded that the stratification criterion of the median of the VAS utility shows no influence in the sense of effect modification on the weighting results. Thus, a relatively robust result can be assumed across all patients regarding the order of the endpoint weighting. On the other hand, the relatively high proportion of inconsistent patients seems remarkable, which is why an attempt was made to analyze the individual inconsistencies using regression calculations despite the relatively small survey group.

### Regression analysis

The relatively strong inconsistency at both the primary and secondary hierarchical levels of the AHP led to the consideration of identifying explanatory variables for this phenomenon. Inconsistency at the individual and group levels arises within the AHP because of the respondents’ failure to comply with transitivity. In the binary logistic regression model patient characteristics such as age, gender, duration of medication and treatment regimen as well as the VAS utility values were included (Table 4). The Variance Inflation Factor (VIF) indicated a low correlation among the independent variables included in the models.

**Table 4:** Logistic Regression Model.

Logistic Regression Model 1^st^ AHP Hierarchic Level (Hypertension Endpoints)											
Model Summary			Omnibus Test			Variable	Regression Coefficient	SE	Significance	OR	CI_95%_
**-2 Log-Likelihood**	**Cox - Snell R²**	**Nagelkerkes R²**	**X²**	**df**	**Significance**	VAS Utilities	-0.045	0.038	0.233	0.956	0.887-1.030
30.495	0.187	0.250	5.395	5	0.370	**Gender**	1.005	0.885	0.256	**2.733**	0.482-15.486
						Age	-0.030	0.069	0.661	0.970	0.847-1.111
						Duration	0.040	0.112	0.721	1.041	0.836-1.295
						**Scheme**	-0.503	1.271	0.693	**0.605**	0.050-7.306
						Intercept	4.612	5.009	0.357	100.718	
**Logistic Regression Model 2**^**nd**^ **AHP Hierarchic Level (Adverse Events)**											
**Model Summary**			**Omnibus Test**			**Variable**	**Regression Coefficient**	**SE**	**Significance**	**OR**	**CI**_**95%**_
**-2 Log-Likelihood**	**Cox - Snell R²**	**Nagelkerkes R²**	**X²**	**df**	**Significance**	VAS Utilities	-0.035	0.044	0.429	0.966	0.887-1.053
24.519	0.358	0.477	11.524	5	0.042	**Gender**	0.607	1.022	0.553	**1.834**	0.247-13.597
						Age	-0.058	0.079	0.464	0.944	0.808-1.102
						**Duration**	0.233	0.146	0.111	**1.262**	0.948-1.680
						**Scheme**	-1.234	1.492	0.408	**0.291**	0.016-5.422
						Intercept	3.987	5.340	0.455	53.980	

As a result, due to the small number of participants (*n* = 26), the corresponding odds ratios are not statistically significant and can therefore be interpreted more as trends. Among the different calculations, the highest R-squared value (calculated here as Nagelkerke’s R-squared for logistic regressions) was achieved in the logistic regression model for consistency in *Adverse events* (second hierarchy level) with 0.477. Thus, it can explain a variance of the dependent variable (consistency) across all independent variables combined (gender, age, duration of therapy, treatment regimen, and VAS utility values) of almost 50%. In the regression model for the first hierarchy level (*hypertension efficacy endpoints*), a R-squared = 0.250 was achieved. Nevertheless, the R-squared is relatively low, and thus the model can explain little of the variance. Regarding the assessment of model quality using the omnibus test of the model coefficients, the regression model for the first hierarchy level is not significant (0.370). Ultimately not all selected variables influence the consistency as a dependent variable, whereas the model for the second hierarchy level achieves significance (0.042).

In the model for the first hierarchy level, only gender (OR = 2.733) and the treatment regimen (OR = 0.605) show a strong influence.

When looking at the model for the second hierarchy level, in addition to gender (OR = 1.834) and the treatment regimen (OR = 0.291), the duration of treatment also impacts consistency (OR = 1.262), albeit to a lesser extent than the first two variables. This can be hypothetically explained by the fact that with longer therapy more experience has been gained regarding drug-induced *Adverse events*. Furthermore, interactions may also occur with combination therapies. Hence, more consistent results are obtained in the survey at the second hierarchy level, although the treatment regimen already had a strong influence at the first hierarchy level. For both hierarchy levels, a more consistent response tendency depending on the male gender is recognizable – albeit less pronounced at the second hierarchy level. The regression analysis was completely exploratory in nature and aimed only at identifying potentially explanatory variables.

### Evaluation of the AHP survey instrument

Just over half of the participants (14/26, 54%) reported the AHP survey to be ‘rather easy’ to complete whilst the remaining participants reported that they found it to be either ‘moderately difficult’ (7/26, 27%) or ‘very difficult’ (5/26, 19%) to complete. Apart from the two participants not included in the AHP survey, all other participants reported no further problems of understanding regarding the survey method, the description of the (sub-)criteria to be considered at the first hierarchy level and at the second hierarchy level, and the assessment of HRQoL. The mean completion time reached 30.1 ± 6.4 min, and thus unexpectedly a relatively homogeneous result.

## Discussion

*Stroke* occupies a distinct role in the prioritization among *endpoints* when compared to *Mortality*. In a best-worst scaling by Aschmann et al. [26] *Stroke* was the most important outcome to avoid among patients with multiple chronic conditions, reflecting its severe impact on HRQoL. However, in this study *Mortality* was excluded. Using a standard gamble approach, Solomon et al. [27], found that a severe *Stroke* was weighted nearly equivalently to death. In contrast, our study did not subdivide the endpoint *Stroke* to ensure comparability with other defined *endpoints*. To our knowledge, this is the first AHP study to examine patient preferences on efficacy and safety endpoints in the pharmaceutical treatment of high blood pressure including next to Morbidity Adverse events and Mortality.

Though AHP can produce robust and valid results for smaller samples [12], our study with 26 evaluated participants is rather small, which limits robust statistical inference especially for regression models and reduces reliability of the subgroups carried out. Furthermore, subgroup analysis depending on socio-economic status would allow for a more extensive analysis on potential impact of this criterion on elicited preferences. The structured interviews as the chosen approach for the administration of the AHP questionnaires enabled personal interaction allowing for clarification of questions and high response rates due to personal touch. Some potential bias due to social desirability or potential interviewer bias cannot be avoided [28]. Another limitation refers to the fact that the study was conducted only in three pharmacies in two German regions and therefore the results may not be generalizable.

AHP is primarily method focused on preference elicitation rather than statistical inference. Previous healthcare AHP studies have used comparable sample sizes (10-30 participants) [13–18] to derive stable group-level weights when consistency ratios are within acceptable limits. On an aggregated level consistency ratios showed acceptable magnitudes, whereas on an individual level consistency ratios partially exceeded consistency ratio thresholds.

Methodological weaknesses of the AHP method are discussed in international literature. Regarding the rating scale used, it has been criticized for not reflecting every realistically possible assessment and that under certain conditions rank reversal can occur [29]. Yet, AHP is considered the most frequently implemented multi-criteria method worldwide [30] and there are recent applications in public-health relevant topics [31–33] and regarding patient-relevant endpoints [34] as well as different treatment strategies in specific conditions [35]. Shafir and LeBoef [36] show that individuals often violate rationality assumptions about logical conclusions, judgments, and choices in their decisions, deviating from the normative model of behavior. They support the criticism of rationality as an axiomatic form of behavior that is now emerging in the social sciences. Other well-established methods like Discrete Choice Experiments (DCEs) often present cognitive challenges for respondents, especially when the respective experiment involves many attributes or when respondents suffer from cognitive impairments. The AHP with its pair-wise comparison and weighting seems to cause less cognitive burden. The cognitive challenges in DCE stem from the cognitive effort required to evaluate parallelly trade-offs between different attributes and make choices, potentially leading to the use of simplifying heuristics and thereby even inaccurate responses [37]. Nonetheless, the high individual inconsistency shown in the present AHP survey may reflect cognitive burden or survey complexity, even though over half of the participants reported that the survey was ‘rather easy’ to complete.

Aggregated patient preferences can be brought in for Health Technology Assessment (HTA) purposes in a positive way. In a recent exploration of the uses of patient preferences in HTA the authors concluded that patient preferences would add most value for informing what and how much matters to patients (e.g., endpoint selection and benefit-risk assessments) [38]. Furthermore, individual patient preferences can be used in the context of shared decision-making (SDM) as an alternative to traditional informed consent procedures [39]. SDM has been proposed as a model for clinical practice, where good communication skills of physicians are integrated with the use of patient decision support tools to explore preferences and make decisions [40]. In our case this translates to an individual approach for deriving patient preferences to decide on different strategies in the treatment of primary hypertension depending on endpoint weighting. AHP can be used on an individual level as a decision supporting tool [23] by modern digital technologies.

AHP can be used to obtain *preferences* of patients suffering from *primary hypertension* for *efficacy* and *safety endpoints*. *Preference* elicitation could provide important information and challenge opinions on the importance of respective *endpoints*, as in the present study the avoidance of *Stroke* was similarly weighted as or even higher than *Mortality*. This highlights the importance of individual *patient preferences* to facilitate tailored therapies for improving benefit. Considering *patient preferences* fosters *patient-centered* approaches and can have even an impact on treatment success.

## Summary

### What is known about this topic:

- Stroke is the most important outcome to avoid among patients with multiple chronic conditions but is usually not compared against mortality in patient preference elicitation studies and medication adverse events are not included in these studies.

### What this study adds:

- The study examines patient preferences on efficacy and safety endpoints in the pharmaceutical treatment of high blood pressure including next to morbidity adverse events and mortality with stroke as an event to be avoided receiving the highest weight, followed by mortality and myocardial infarction.
- The results of the patients’ preference elicitation in hypertension can be used to tailor individual treatment within Shared decision-making or for Health Technology Assessment purposes as well.
- The Analytical Hierarchy Process (AHP) survey is feasible and accepted by patients with primary hypertension regarding the assessment of different endpoints of their treatment.

## Supplementary information

ESM Figure

ESM text 1

ESM text 2

## Data Availability

Data and supporting materials are available upon request.
