# Supplementary material for: How do patients with primary hypertension assess different endpoints of their treatment? a survey using analytic hierarchy process
Source: J Hum Hypertens. 2026 Mar 23;40(4):333–41. doi: 10.1038/s41371-026-01135-8 (PMC13068516; doi:10.1038/s41371-026-01135-8)
Supplement: Supplementary file 1 — ESM Figure [file 41371_2026_1135_MOESM1_ESM.docx]

ESM Figure 1: AHP-Rating

Caption: The figure shows the AHP rating-scheme with its nine ratings and the interpretation behind each rating.
