# Supplementary material for: How do patients with primary hypertension assess different endpoints of their treatment? a survey using analytic hierarchy process
Source: J Hum Hypertens. 2026 Mar 23;40(4):333–41. doi: 10.1038/s41371-026-01135-8 (PMC13068516; doi:10.1038/s41371-026-01135-8)
Supplement: Supplementary file 2 — ESM text 1 [file 41371_2026_1135_MOESM2_ESM.docx]

**Analytic Hierarchy Process (AHP): Consistency and Weights**

*Consistency*

For each matrix of pairwise comparisons, the AHP provides a measure of consistency to show if each pairwise comparison is logically sound with regard to the remainder of the comparisons [1, 2]. For example, in this application one of the matrices of pairwise comparisons includes the comparison of the importance between stroke and myocardial infarction, between myocardial infarction and heart failure, and between stroke and heart failure. If a participant had indicated to consider stroke to be strongly more important than myocardial infarction, myocardial infarction to be strongly more important than heart failure, and heart failure to be strongly more important than stroke, the participant would be clearly inconsistent in these judgments [3]. The degree of inconsistency is based on the λ_max_, which is the maximum eigenvalue of the matrix of pairwise comparisons [2]. Normalizing this measure by the number (n) of criteria to compare, Saaty [1, 4] defines the consistency index (C.I.) as:

1. C.I. = (λ_max_ – 1)/ (n-1)

For each matrix of pairwise comparisons, 500 random matrices are generated and their mean C.I. value, called the random index (R.I.) is computed. The consistency ratio (C.R.) indicates how far the pairwise judgments deviate from a purely random matrix of pairwise comparisons, and is defined as [1, 2]:

1. C.R. = C.I./R.I.

Saaty’s rule of thumb is that ten per cent of the inconsistency of the random matrix is allowed. This implies that a value of the C.R. ≤ 0.1 can be considered acceptable and generates plausible outcomes [5]. In case of higher inconsistency, the decision makers are urged to check for accidental mistakes and to reconsider their pairwise comparisons, until the consistency measure will be below the threshold indicated [6].

*Weights*

In case of acceptable degrees of inconsistency, weighting factors are to be calculated. If judgments are not fully consistent, there are different ways to estimate the weights of the criteria. The principal right eigenvector approach is recommended by Saaty [5]. In this approach, the maximum eigenvalue of the matrix of pairwise comparisons (a_ij_) will be used to estimate the weights (wi) of the criteria:

1. w_i_ = (∑n j=1 a_ij_ * w_j_) / λ_max_ for all i = 1,2,…,n.

This eigenvector method can be interpreted as being an averaging process by which the final weights are the average of all possible ways of comparing the importance of the criteria. In case of an acceptable degree of inconsistency, the weight factors assigned to the outcome measures are plausible to represent the relevance of each of these outcome measures to the patients.

*Group average*

When calculating a group average to reflect the opinion of the group as a whole, the use of the geometric mean of all pairwise comparisons is recommended [7]. Consequently, when the AHP supports a group of x (x = 2,3…) members, the aggregated pairwise comparisons are computed by the geometric mean of the individuals’ comparisons a_ij_:

1. a_ij_ = (a_ij_(1) * a_ij_(2) * … * a_ij_(x))^(1/x)^ for all i,j = 1,2,…,n.

Weights and consistency ratios are then calculated for the group as a whole. At the group level, the consistency ratio is derived from the geometric means of the pairwise comparisons, again permitting ten per cent of the inconsistency of the random matrix. If the differences between weights assigned by the individual group members are to be analyzed, these weights can be calculated based on each group member’s pairwise comparisons.

**References**

1. Saaty TL. A scaling method for priorities in hierarchical structures. Journal of mathematical psychology. 1977;15(3):234-81. <https://doi.org/10.1016/0022-2496(77)90033-5>

2. Dolan JG, Isselhardt BJ, Cappuccio JD. The Analytic Hierarchy Process in Medical Decision Making:A Tutorial. Medical Decision Making. 1989;9(1):40-50. doi: 10.1177/0272989x8900900108. PubMed PMID: 2643019. <https://doi.org/10.1177/0272989x8900900108>

3. Saaty TL. RANK GENERATION, PRESERVATION, AND REVERSAL IN THE ANALYTIC HIERARCHY DECISION PROCESS. Decision Sciences. 1987;18(2):157-77. doi: <https://doi.org/10.1111/j.1540-5915.1987.tb01514.x>.

4. Saaty TL. Group Decision Making and the AHP. In: Golden BL, Wasil EA, Harker PT, editors. The Analytic Hierarchy Process: Applications and Studies. Berlin, Heidelberg: Springer Berlin Heidelberg; 1989. p. 59-67. <https://doi.org/10.1007/978-3-642-50244-6_4>

5. Saaty TL. Highlights and critical points in the theory and application of the Analytic Hierarchy Process. European Journal of Operational Research. 1994;74(3):426-47. doi: <https://doi.org/10.1016/0377-2217(94)90222-4>.

6. Harker PT. Derivatives of the Perron root of a positive reciprocal matrix: with application to the analytic hierarchy process. Applied Mathematics and Computation. 1987;22(2-3):217-32. <https://doi.org/10.1016/0096-3003(87)90043-9>

7. Forman E, Peniwati K. Aggregating individual judgments and priorities with the analytic hierarchy process. European journal of operational research. 1998;108(1):165-9. <https://doi.org/10.1016/S0377-2217(97)00244-0>
