# Supplementary material for: How do patients with primary hypertension assess different endpoints of their treatment? a survey using analytic hierarchy process
Source: J Hum Hypertens. 2026 Mar 23;40(4):333–41. doi: 10.1038/s41371-026-01135-8 (PMC13068516; doi:10.1038/s41371-026-01135-8)
Supplement: Supplementary file 3 — ESM text 2 [file 41371_2026_1135_MOESM3_ESM.docx]

Online Resource 2: Description of efficacy and safety endpoints

**Heart attack:** In a heart attack (myocardial infarction) one part of the heart is suddenly not sufficiently supplied with oxygen anymore, which can lead to permanent damage of the heart muscle tissue. A myocardial infarction (the word myocard is of Greek origin and means “heart muscle”, the word infarction is from the Latin for “crammed in”) is most commonly caused by a small blood clot that has blocked one or several of the blood vessels of the heart. Typical symptoms of a heart attack include sudden severe chest pain, which often radiates to the left arm, the upper abdomen and the lower jaw, nausea, circulatory problems and possibly collapse, anxiety and breathlessness. But other, less typical symptoms like abdominal or back pain can also be signs of a heart attack”.

**Stroke:** A stroke, also known as a cerebral stroke or apoplexy (from Greek apoplessein = to strike down), is an acute condition in which the brain no longer receives sufficient oxygen. In most cases, the cause is a blood clot that blocks blood vessels in the brain. Less commonly, a cerebral hemorrhage can also lead to a stroke. Depending on the affected brain region, symptoms may include paralysis of one side of the body or parts thereof, facial nerve deficits, visual disturbances, balance problems, and severe speech impairments. A stroke is a medical emergency: The affected brain regions must be resupplied with oxygen as quickly as possible to prevent further brain tissue damage. The risk of a stroke is higher in older individuals, those with high blood pressure, or those with chronic vascular calcification.

**Acute heart failure:** In heart failure (cardiac insufficiency), the heart as a whole or parts of it are too weak to pump sufficient blood into the circulatory system, resulting in poor supply to organs and muscles. Heart failure can severely limit a person's physical capacity, to the point where any movement causes shortness of breath, pain, or other symptoms. Some individuals experience acute, short-term heart failure as a result of a heart attack or cardiac arrhythmia. Others are affected by a chronic form, triggered, for example, by persistent high blood pressure or damage to the small blood vessels that supply the heart with blood. Acute heart failure (decompensation), regardless of its cause, constitutes a medical emergency, like a heart attack or stroke, and requires inpatient hospital treatment. In addition to symptomatic treatment and stabilization of the circulatory condition, further therapy depends on the underlying cause of the condition.

**Adverse events:**

**Dyspnea** or shortness of breath is the uncomfortable feeling of not being able to breathe well enough.

**Edema** means swelling. It arises when part of the body becomes swollen because fluid gathers in the tissue. It is most common in the arms and legs.
